# Adaptation of the Motivational Interviewing Skills Code to Identify Client Language Predicting Reduced Opioid Use Risk and Increased Use of Alternative Pain Care Strategies in Veterans

**DOI:** 10.3390/mps8060149

**Published:** 2025-12-08

**Authors:** Brian Borsari, Catherine Baxley, Benjamin O. Ladd, Joannalyn Delacruz, Kristina M. Jackson, Theodore Fetterling, Kyle J. Self, Shahrzad Hassanbeigi Daryani, Karen H. Seal, Jennifer K. Manuel

**Affiliations:** 1 San Francisco Veterans Affairs Health Care System, San Francisco, CA 94121, USA; catherine.baxley1@va.gov (C.B.); jbd95@cornell.edu (J.D.); theodore.fetterling@va.gov (T.F.); karen.seal@va.gov (K.H.S.); jennifer.manuel@va.gov (J.K.M.); 2 Department of Psychiatry and Behavioral Sciences, University of California, San Francisco, CA 94143, USA; 3 Department of Psychology, Washington State University Vancouver, Vancouver, WA 98686, USA; benjamin.ladd@wsu.edu; 4 Department of Psychiatry, Rutgers Robert Wood Johnson Medical School, Rutgers Addiction Research Center (RARC), Rutgers the State University of New Jersey, New Brunswick, NJ 08901, USA; kristina.jackson@rutgers.edu; 5 Department of Public Health Sciences, University of Miami, Miami, FL 33146, USA; k.self@miami.edu; 6 Rush Medical College, Chicago, IL 60612, USA; shahrzad_hassanbeigidaryani@rush.edu; 7 Department of Medicine, University of California, San Francisco, CA 94143, USA

**Keywords:** opioid, pain, primary care, veterans, motivational interviewing, collaborative care

## Abstract

Objective: Motivational Interviewing may be an ideal communication style to use in conjunction with Collaborative Care to address opioid risk, as it can facilitate the discussion of alternative pain care strategies (APCSs) that are pharmacological (APCS-P; e.g., the use of non-opioid pain relievers) or non-pharmacological (APCS-NP; e.g., yoga). This study developed and piloted a coding system (MI Skills Code–APCS) for these discussions. Method: Sessions (*n* = 119) from a completed randomized controlled trial comparing Collaborative Care Motivational Interviewing (CCMI) or Attention Control Psychoeducation (ACP) delivered by care managers over 12 weeks to veterans with chronic pain and high-risk opioid use enrolled in VA primary care (*N* = 44). Results: Coders were able to reliably code the client utterances related to APCSs in the sessions (ICCs = 0.58–0.81). The APCS-P and APCS-NP codes were positively correlated with each other. There were two significant relationships between the MISC-APCS codes (motivational states) and the pain interference and endorsement of non-pharmacological pain care goals at 20-week follow-up. Conclusions: The MISC-APCS has promise as a coding system that can reliably record client utterances regarding different types of pain care strategies. These utterances may be associated with post-treatment reports of pain and efforts to reduce opioid risk. The rapid development of artificial intelligence applications to healthcare can utilize this coding system to assist with the assessment and treatment of chronic pain.

## 1. Introduction

In 2017, the US government declared opioid use a public health emergency [1]. Military veterans have been disproportionately impacted by the opioid crisis [2,3,4]. Collaborative Care, which uses nurse care managers to improve communication between patients and their clinicians, has been shown to improve pain management in primary care patients [5,6]. Motivational Interviewing [7] may be an ideal communication style to use in conjunction with Collaborative Care to address opioid risk, as it can facilitate the discussion of directly reducing opioid risk (e.g., opioid taper) or the adoption of alternative pain care strategies (APCSs; e.g., yoga, acupuncture, the use of non-opioid pain relievers). MI is a directive, non-judgmental, patient-centered approach intended to enhance patients’ intrinsic motivation to change by exploring and resolving ambivalence about a specific behavior [8]. MI has strong evidence for the treatment of substance use disorders [9,10,11] and preliminary evidence for efficacy in addressing risky opioid use in chronic pain patients in an outpatient setting [12]. However, nothing is known about how MI works to facilitate the reduction in opioid use or the adoption of APCSs in veterans.

MI theory and research posit that patient speech expressing a commitment to change (“Change Talk”) is associated with improved patient outcomes, and speech opposing change (“Sustain Talk”) is related to poorer outcomes [8,10,11]. The Motivational Interviewing Skills Code 2.5 (MISC 2.5) [13] is the current state-of-the-art client language measurement system in MI; however, it only captures language explicitly linked to a single target behavior and a specified change direction (e.g., opioid use reduction). Thus, a considerable amount of client in-session verbal behavior is grouped into a follow/neutral (FN) category for client utterances that address anything other than opioid use, e.g., “I like to take aspirin when I am sore”) and may be lost with the MISC 2.5. An “utterance” was defined as a single, discrete statement that expresses one idea, intention, or piece of information [13]. Behavioral economic theory and its focus on the appeal and engagement in a variety of activities may hold promise in this context (see [14]). Specifically, given that MI is often used with veterans without an immediate desire to reduce opioid use or risk (the target behavior), adopting APCSs may be considered, which in turn may result in decreased pain and thus lessen the need for opioids. Considering motivational state (approach, neutral/ambiguous, avoid) for not only the target behavior but also APCSs independently has the advantage of not presuming the same desired direction of change for all clients. Coding behavior and motivation independently address this issue and allow for identifying how much language is “on target” even if motivationally neutral (e.g., reporting on opioid use), which would be considered follow/neutral under the MISC 2.5. To our knowledge, this is the first study to examine client language as a predictor of changes in opioid use and/or the adoption of APCSs.

This proof-of-concept study utilized sessions from a completed randomized controlled trial with chronic pain patients in VA primary care [15] to provide an ideal opportunity to explore client speech during the discussions of APCSs. In the Opportunities for Pain Innovation (OPTI) trial, veterans exhibiting “high-risk” opioid use behavior were randomized to receive four sessions of either Collaborative Care Motivational Interviewing (CCMI) or Attention Control Psychoeducation (ACP) from care managers over 12 weeks. Veterans in both the CCMI and APC groups reported significant decreases in scores on the Addiction Behavior Checklist, Current Opioid Misuse Measure, and completion of APCSs [16]. In addition, veterans in the CCMI group reported implementing more complementary integrative health (CIH) goals (e.g., yoga) than those in the ACP group.

## 2. Materials and Methods

**Figure 1:**
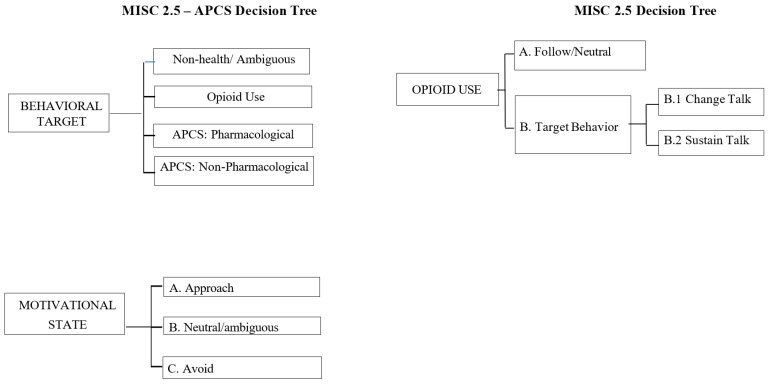
Comparison of Motivational Interviewing Skills Code (version 2.5; MISC 2.5) and alternative pain care strategies (APCSs).

This study aimed to explore the feasibility of capturing novel theoretically driven client language definitions. First, we sought to develop a novel MI fidelity coding instrument to reliably capture change language about APCSs (currently not coded by the MISC 2.5). As can be seen in Figure 1, this enhanced coding system, the MISC-APCS, is innovative in that it explores the motivational state (avoid, approach, and neutral/ambiguous language) related to three behaviors: APCS-Target Behavior (APCS-TB; i.e., using opioids), APCS-Pharmacological (APCS-P; i.e., using non-opioid medication), and APCS-Non-Pharmacological (APCS-NP; i.e., actions and behaviors that served as competing sources of reinforcement away from the target behavior of opioid use). The MISC-APCS coding system used a parallel decision-making process for assigning an individual code to different topics (APCS-TB, APCS-P, APCS-NP). In other words, in addition to capturing the change language of the identified target behavior (opioid use), the proposed coding system also explored the motivational state (approach, neutral, avoid) of client language related to APCS-P and APCS-NP. Second, we conducted the preliminary examinations of the MISC-APCS to test client language as a predictor of response to the CCMI and ACP sessions at the 20-week follow-up (to best capture behavior change following CCMI and ACP). We hypothesized that the discussion of APCSs would be associated with (a) a decreased opioid risk and (b) an increased number of APCSs.

### 2.1. Design

Sessions were from OPTI, a completed randomized controlled trial assessing chronic pain in VA primary care [15,16]. Patients enrolled in the trial were prescribed opioid pain medications and exhibited at least one “high-risk” opioid use behavior (e.g., obtaining early opioid refills). Veterans received a pain care assessment and planning session to develop personal pain care goals at baseline by one of the study clinicians and were then randomized to CCMI (n = 40) with 4 telephone MI sessions or ACP (n = 36) with 4 neutral telephone sessions (reviewing opioid risks and checking in with the patients) over 12 weeks. Outcomes for participants in the CCMI and ACP groups were assessed by phone at baseline, post-intervention (12 weeks), and at follow-up (20 weeks) by study staff masked to group assignment.

### 2.2. Participants

Our inclusion criteria included adult veterans (≥18 years) with chronic pain (>6 months duration) currently enrolled in VA primary care and receiving long-term opioid therapy, defined as being prescribed opioids (including Tramadol) for at least the past 90 consecutive days [17,18]. In addition, veterans had to demonstrate one or more of the following a priori proxies for “high-risk” opioid use (based on VA administrative data): (a) higher-dose opioid therapy (≥45 milligram morphine equivalent daily dose [MEDD]) [19]; (b) a score of ≥ 9 on the Current Opioid Misuse Measure (see below); (c) co-prescription of benzodiazepines [20]; (d) at least one early opioid refill in the last 12 months (prior to refill date); (e) current depression, post-traumatic stress disorder (PTSD), or other anxiety disorders from chart review [3]; and/or (f) evidence of current or former mild-to-moderate drug and/or alcohol abuse or dependence (e.g., positive urine drug screen, chart review, etc.) [21]. Exclusion criteria were as follows: (a) inability to comprehend English; (b) prescribed opioids in the context of palliative or hospice care; (c) serious untreated mental illness (i.e., psychosis, bipolar disorder, or severe substance abuse or dependence); or (d) active suicidal/homicidal ideation.

### 2.3. Measures

#### 2.3.1. Demographics

Participants’ sociodemographic characteristics were assessed at baseline and included age, gender, race/ethnicity, education, history of homelessness, current employment status, and VA service connection for health problems or disability.

#### 2.3.2. Opioid Use

Morphine equivalent daily dose (MEDD) at baseline was calculated using procedures outlined by the Centers for Disease Control [22].

#### 2.3.3. Opioid Risk

A co-primary outcome was the Current Opioid Misuse Measure (COMM) [23], a 17-item self-report questionnaire that tracks current aberrant opioid-related behaviors. Items are rated from 0 (“never”) to 4 (“very often”) with a total maximum score of 68. A cutoff score of ≥ 9 is considered positive (a sensitivity of 0.77 and a specificity of 0.66). In this sample, α = 0.86 for the 17 items. The Addiction Behavior Checklist (ABC) [24] is a 20-item clinician-rated checklist to track opioid-related addictive behavior and was administered by study PCPs. A cutoff score of ≥ 3 showed optimal sensitivity and specificity in determining aberrant opioid-related behavior.

#### 2.3.4. Pain

The other co-primary outcome was the Pain Interference Scale of the Brief Pain Inventory (BPI) [25]. The BPI is an 11-item measure of two domains: pain severity and interference with functioning. The average of the items of each domain (scored from 0 to 10) was used to determine an overall continuous pain severity and interference score. The BPI has demonstrated internal consistency and test–retest reliability in previous trials (see [25]). In this study, internal consistency was good for both severity (α = 0.87) and interference (α = 0.90).

#### 2.3.5. Pain Care Plan Goals

To evaluate the number of personal pain management goals established at 20 weeks, items in each Pain Care Plan were extracted and classified using a hierarchical taxonomy developed for this study [15,16]. For this study, APCS-TB included tapered or reduced use of opioids, APCS-P included non-opioid-based pharmacological substances (e.g., topical ointments), and the APCS-NP included activities that were complimentary and integrative to health, such as CIH (e.g., yoga, acupuncture) or non-CIH (e.g., physical therapy, walking).

### 2.4. Study Interventions

#### 2.4.1. Collaborative Care with MI (CCMI)

As detailed previously [15,16], the CCMI consisted of four < 30 min sessions conducted by care managers. Care managers (CMs; n = 3) were clinical psychologists, postdoctoral fellows, or psychology research staff who had been trained to utilize MI to further align Pain Care Plans (developed during the initial primary care visit), with participants’ personal values and goals. The first session was conducted in-person (immediately following the initial primary care visit at 4 weeks), and subsequent sessions were conducted by telephone at 6, 8, and 12 weeks. These telephone sessions assessed pain and discussed progress toward achieving SMART goals in their Pain Care Plans as well as behaviors to decrease opioid risk and increase the use of non-opioid alternatives for pain management. CMs did not make changes to the goals as they were not medical providers and focused on the goals that were most relevant to the veteran rather than forcing the veteran to discuss each goal on the plan. CMs used MI skills (e.g., reflective listening) and the Readiness Ruler to evoke change language about opioid risk reduction and the adoption of non-opioid alternatives. CMs received ongoing supervision, and their sessions were monitored for treatment fidelity.

#### 2.4.2. Attention Control Psychoeducation (ACP)

The same CMs also provided the ACP sessions to participants randomized to this condition. ACP sessions were shorter in length and consisted of neutral psychoeducation about opioid safety. CMs did not administer the Readiness Ruler nor used MI-consistent skills when providing standard VA education about chronic pain and opioid safety, answering participants’ questions, and reviewing upcoming study visits.

### 2.5. Within-Session Mechanisms of Behavior Change

#### 2.5.1. Motivational Interviewing Skills Code Version 2.5 [13]

Sessions were transcribed and coded with the MISC 2.5 to determine the utility of adding APCS codes. Although the MISC 2.5 measures 19 categories of MI behaviors (e.g., open questions, simple reflections), we focused on global ratings that are designed to capture the CM’s interaction with the veteran throughout the entire session. The 6 global ratings are empathy, acceptance, direction, autonomy support, collaboration, and evocation. There is one global rating for veteran self-exploration. These ratings are scored on a 5-point Likert scale ranging from 1 (low) to 5 (high).

#### 2.5.2. Motivational Interviewing Skills Code–APCS (MISC-APCS) Coding System

The current MISC 2.5 coding system was adapted in two ways (see Figure 1). First, codes were developed that assessed all in-session veteran language related to the target behavior of opioid use (APCS-TB), using non-opioid pharmacological medications (APCS-P) or non-pharmacological behaviors (APCS-NP) that can impact the degree of reinforcement of opioid use. Utterances that were not related to opioid use, APCS-P, or APCS-NP were not assigned a code (No Code). Second, motivational state of opioid use, APCS-P, or APCS-NP utterances were rated based on whether the veteran was likely to approach or avoid the behavior being discussed, such as choosing to take ibuprofen to manage pain (APCS-P approach). Motivational state can also be rated as neutral/ambiguous if approaching or avoidance is not evident in the statement (e.g., “Ibuprofen pills are shaped weird”). It is important to note that the MISC-APCS was designed to significantly overlap with the MISC 2.5 definition of change talk and sustain talk of the target behavior. Specifically, TB Avoid is similar to the MISC change talk, and the TB approach was conceptualized similarly to the MISC sustain talk. Previous research coding alcohol-focused interventions in college students has found high correlations between approach codes and change talk and avoid codes and sustain talk [14]. The added value of the MISC 2.5-APCS was in the P and NP codes, much of which likely would fall under the MISC follow/neutral code when not explicitly linked to opioid use. In this way, the MISC-APCS was not designed as distinct from the MISC, but rather to augment with more nuanced definitions of non-target language.

### 2.6. Coder Training and Data Collection

Weekly meetings were used to resolve discrepancies between the three coders, discuss coding issues that came up during the previous week, and to add coding “decision rules”, when needed, to the project log to enhance subsequent consistency among coders.

The coding of in-session language occurred in three phases. First, coders were trained on the coding process and system. A set of audiotapes from the parent study, consisting of sessions from participants for whom no follow-up outcome data were available (n = 93), were used for training purposes. Second, all transcripts were parsed prior to being coded, which involves manually marking up transcripts to divide lengthy statements into utterances, each of which were eventually assigned a distinct behavior code. After the team was familiarized with the parsing procedures, they then coded a subset of training sessions as a group and discussed any differences in coding to establish consensus. Coding was conducted while reading the transcript and listening to the session audio recording. Codes were extracted by having the coders rate the MISC 2.5 globals and therapist behaviors, then focus on the novel MISC-APCS client utterances (APCS-TB, APCS-P, APCS-NP). Data collection commenced with a team of three coders who worked independently, double-coding a random subsample of ~20% of sessions to establish interrater reliability.

### 2.7. Establishing Coding Dataset

As one of the purposes of this study was to develop novel client language codes (APCS-TB, APCS-P, APCS-NP), the coding definitions continued to be updated and altered based on discussions during weekly coder meetings after training was completed. Based on coder calibration needs, we decided to consider the first six weeks of data collection as the pilot data that were not used for the final analyses (*n =* 20). All sessions were coded regardless of condition; however, sessions that were less than 10 min in duration (*n* = 125) were not included in the final dataset as these sessions were primarily a brief check-in with the veteran and global ratings cannot be reliably coded in sessions under 10 min [26]. As the amount of veteran language is limited, inclusion of these check-in sessions could unduly influence the percentages of veteran language in discussions about pain and opioid use, which is our measurement strategy. The final dataset consisted of 119 sessions from 44 unique individuals (37 in CCMI; 7 in ACP). Chi-square tests and t-tests comparing participants with coded and uncoded sessions did not reveal any significant differences in demographic or outcome variables.

### 2.8. Analysis Plan

To describe client language, we examined the MISC 2.5 globals and MISC-APCS codes descriptively. To evaluate the reliability of the MISC-APCS coding system, we computed interrater agreement for the MISC-APCS using two-way random, absolute-agreement, single-measure ICCs. We then converted raw counts of client codes to proportions of total client utterances to account for client verbosity. Correlations among proportions were conducted, followed by a series of regressions to predict outcomes at 20 weeks (using baseline values as a covariate) from client language variables, utilizing separate models for each outcome (MEDD, COMM, BPI [severity, interference], and proportions of APCS-P and APCS-NP).

## 3. Results

### 3.1. Preliminary Analyses

Of the 119 sessions across 44 participants, 22 (18%) were double-coded for reliability purposes. In the dataset, there were 12,016 veteran utterances across all 119 individual session tapes [M (SD) = 101.0 (42.8) client utterances per session, range = 25–355]. For our primary analyses, we utilized the individual-level data aggregated across the 119 coded sessions.

### 3.2. Descriptive Information

Descriptive statistics behavior counts as well as continuous outcome variables are reported in Table 1. ICCs for global codes indicate fair interrater reliability for acceptance (ICC = 0.43) and autonomy support (ICC = 0.43); ICCs for all other global codes were poor (i.e., <0.40). However, this may represent an overly conservative estimate of reliability due to concerns of restriction of range (e.g., less than 5% of all global ratings were 1 s or 2 s, 64% or more of ratings for each global were 4 s or 5 s except for self-exploration). Thus, we also report percent agreement within 1. On this measure, the raters demonstrated high agreement on the global ratings, with three of the global ratings (i.e., accept, empathy, and autonomy support) displaying 100% agreement within one rating. The remaining global ratings ranged from nearly 77% to 91% agreement within one rating. Given the mixed reliability estimates for global codes, we included the descriptive for these variables, but did not examine in any additional analyses. As determined by Cicchetti’s [27] criteria, all APCS client codes demonstrated “fair” to “excellent” intraclass correlation coefficients (range = 0.46–0.82, Table 1).

### 3.3. Correlations Between MISC-APCS Client Codes

Correlation coefficients between proportions of language codes are reported in Table 2. Regarding APCSs, each of the three APCS-NP codes displayed low-to-moderate correlations (*r*s = 0.35–0.69) with each other, according to Mukaka’s [28] criteria (very high: *r* = 0.90 to 1.0; high: *r* = 0.70 to 0.90; moderate: *r* = 0.50 to 0.70; low: *r* = 0.30 to 0.50; negligible *r* = 0.00 to 0.30). Similarly, low-to-moderate positive correlations (*r*s = 0.38–0.56) were observed among all three APCS-P codes. For the opioid use target behavior (TB) codes, each TB code displayed low-to-moderate positive correlations with each other (*r* = 0.27–0.66). Taken together, the pattern in the APCS codes indicates that approach, avoid, and neutral statements seem to appear together when discussing the different pain care strategies, and others do not (e.g., APCS-TB-N and APCS-TB avoid).

Overall, the different APCS domains exhibited positive correlations with each other. Low positive correlations (*r* = 0.33–0.45) were observed between the APCS-NP approach and the following codes: APCS-P avoid, APCS-TB approach, APCS-TB neutral, and APCS-TB avoid. APCS-NP neutral displayed low positive correlations (*r* = 0.37–0.46) with the following codes: APCS-P neutral, APCS-P avoid, and APCS-TB neutral. APCS-NP avoid was associated with low positive correlations (*r* = 0.31–0.44) for the following codes: APCS-P approach, APCS-P neutral, APCS-P avoid, and APCS-TB neutral. Similarly, APCS-P neutral displayed low positive correlations (*r* = 0.37–0.48) with the following codes: APCS-TB approach, APCS-TB neutral, and APCS-TB avoid. APCS-P avoid was associated with moderate positive correlations for APCS-P approach (r = 0.53) and APCS-TB avoid (r = 0.50). Lastly, No Code was associated with low-to-high negative correlations with all of the codes (−0.38 to −0.80).

### 3.4. MISC-APCS Client Language Predicting Outcomes

Linear regressions of in-session client codes predicting outcomes revealed two models with significant findings: a greater proportion of APCS-TB approach utterances was associated with higher BPI pain interference (*B* = 35.4, SE = 17.6, *t* = 2.01, *p* = 0.05, B = 0.23, *R*^2^ = 0.52) and a lower proportion of APCS-P approach utterances was associated with greater ACPS-NP goals (*B* = −21.8, SE = 9.86, *t* = −2.21, *p* = 0.03, B= −0.32 *R*^2^ = 0.10). Although not significant, likely due to the relatively small sample size, standardized betas from ten additional models approximated these values (ranging from 0.20 to 0.26 in magnitude; most of these were for alternative pain care strategies as an outcome). Logistic regressions of the within-session client codes predicting dichotomous outcomes at the 20-week follow-up (31 of 41 participants were below a COMM cutoff of ≥9; 26 of 34 participants were below an ABC cutoff of ≥3) were not significant and exhibited low effect sizes. McFadden’s pseudo R-squared values ranged from 0.001 to 0.09, corresponding to very-low-to-low fit. With N = 34 and N = 41, however, some of the models were unstable (and odds ratios exhibited wide confidence intervals).

## 4. Discussion

Chronic pain remains a significant problem in the US population, and the movement away from the prescription of opioids to address this issue has resulted in many alternate pain care strategies. These strategies are often presented in the context of discussions about pain between patients and their providers, often in the primary care setting. The systematic analysis of these conversations and subsequent clinical outcomes would provide valuable guidance on how to effectively discuss non-opioid, non-pharmacological pain care strategies in a way that facilitates their adoption into everyday life. This study detailed the development and preliminary testing of such a coding system, the MISC-APCS.

These preliminary findings suggested that different types of alternative pain care strategies, whether pharmacological, non-pharmacological, or focused on changes in the use of opioids (e.g., reducing dosage, etc.), can be reliably coded in sessions. Therefore, the use of this system may be indicated in settings in which providers are discussing a variety of strategies with their patients. One consideration is that there was a low frequency of many of the codes, which was not surprising as the coaching sessions that were coded were collaborative and explored aspects of the veterans’ experience of pain with a gentle exploration of pain care strategies as indicated. This structure did not always result in the exploration of all pain care goals and as a result may have precluded client utterances about them. Indeed, there was a large amount of No Code client language in each session, which coders were able to reliably differentiate from APCS utterances. Future uses of the MISC-APCS may provide useful information for determining contexts where higher levels of APCS codes may be present, such as setting in more focused conversations about pain care alternatives.

There were two significant findings linking in-session proportions of client utterances to subsequent outcomes, and both were in intuitive directions that may reflect client utterances reflecting personal impairment from pain and specific pain care goals. First, veterans with a greater proportion of utterances about opioid use reported greater pain interference. This finding indicates that opioids are likely to be a topic of conversation with veterans whose lives are being impacted by chronic pain. Second, veterans whose sessions had a lower proportion of utterances expressing desire to use pharmacological APCSs had more non-pharmacological pain care strategies on their Pain Care Plans. Overall, however, APCS utterances were not significantly related to a wide range of outcomes in this development pilot study. There are several reasons to explain the lack of observed effect. First, although effect sizes from correlations are accurate, the regression analyses may have been underpowered to detect effects. Second, client language evoked during coaching sessions about APCSs may not be predictive of changes in opioid risk and pain outcomes. In the MI literature, there have been findings that change talk is not predictive of subsequent behavior change; instead, sustain talk or the ratio of change talk to sustain talk has been linked to outcomes (see [10,11]). It has been posited that, in MI sessions, an empathic clinician may evoke unrealistic or disingenuous change language [7,29]. The same dynamic may be present when coding APCSs. Third, the coaching sessions may not have been where the majority of predictive client language associated with outcomes occurred. Instead, it may have been evoked during the visits with the study PCPs, common to participants in both groups (1 h at baseline and 30 min at 12 weeks). During these sessions, which were not recorded, initial Pain Care Plans with APCSs were developed and then revisited. Additional tests of the MISC-APCS to evaluate language during such interactions could be beneficial to further validate this system as well as provide valuable information on relationships between in-session language and pain outcomes.

These findings should be considered in the context of several limitations. First, the final sample size (N = 44 participants, 119 sessions) limited our ability to detect moderate associations between in-session language and outcomes. Initial power analyses assumed a sample size of N = 76, but the final sample size was reduced due to a variety of reasons, including exclusion of sessions shorter than 10 min from analyses and corrupted or unintelligible recordings. Replication on larger samples would enhance confidence in the reported results of this preliminary trial. Second, we did not code client language using the MISC 2.5 definitions of change talk, sustain talk, and follow/neutral, precluding analyses regarding their association with APCS codes. Third, some of the interrater reliability estimates were modest, and thus conclusions drawn from this preliminary investigation for the relationships between novel language codes and post-intervention outcomes should be considered with caution. This was especially the case with the global codes, on which there was high agreement but low reliability, indicating a lack of range in the ratings. Finally, veterans who had proxies for being high risk with respect to opioids were challenging to recruit [15,16]; therefore, the conversations that were recorded are likely not representative of all veterans with high-risk opioid use.

There are several promising directions for research utilizing the MISC-APCS system. First, replication in larger samples with longer follow-up periods would enhance confidence in the reliability of the coding system and the validity of the APCS codes. More intensive training using representative practice session, frequent meetings of the coding team to discuss rating discrepancies that inform iterative and detailed decision rules, and regular reliability checks can enhance the reliability of behavior and global codes [30]. Second, if the APCS codes are predictive of subsequent changes in opioid use and alternative pain care strategy initiation and maintenance, the relationship between therapist skills (e.g., reflections, open-ended questions, affirmations) and client utterances would be valuable to explore. The identification of clinician skills that are most likely to enhance APCS approach language would be valuable for providers and supervisors to incorporate in their clinical practice and may inform how to best structure interactions with their patients. For example, opioid use could first be discussed, and evoked patient curiosity or interest for alternative options (TB neutral or TB avoid language) could serve as a bridge to a personally relevant discussion of pharmacological and non-pharmacological options to address pain, in which approach language could be strategically evoked. The iterative influence of theory informing process coding, which in turn informs training and supervision and clinical practice, is a precedent in the construction of change talk in MI (see [31]). Third, as confidence grows in the preliminary findings reported here, clinician skills that can evoke client language predictive of the adoption of APCSs and/or reduction in opioid use can also be incorporated into prompts used by chatbots to guide interactions with users [32,33]. In this way, chatbots could be used to evoke, reflect, and affirm APCS approach language in patients.

## 5. Conclusions

This study sought to develop and provide a preliminary evaluation of whether a coding system can capture conversations about opioid use and alternative pain care strategies. These initial findings indicate that different categories of client utterances during these conversations can be reliably coded, and that certain client utterances may be predictive of subsequent changes in pain interference and the development of specific goals to reduce pain and opioid risk. These findings, while suggestive, require replication in larger samples in order to enhance confidence that client utterances are an appropriate focus in the efforts to enhance the effectiveness of patient and provider conversations about opioid risk.

## Figures and Tables

**Table 1:** Descriptive information of the within-session global ratings and behavior counts (n = 119 sessions) and outcome variables at 20-week follow-up (ns = 34–44).

MISC-APCS Code	M	SD	ICC	95% CI		% Agreement Within 1
				LL	UL	
Global Rating (1–5 range)						
Accept	4.35	0.54	0.43	0.04	0.71	100.0
Empathy	4.28	0.60	0.37	−0.05	0.68	100.0
Direction	4.33	0.52	N/A *			77.3
Autonomy Support	3.81	0.56	0.43	0.01	0.72	100.0
Collaboration	3.85	0.51	0.09	−0.32	0.48	81.8
Evocation	3.76	0.48	0.03	−0.41	0.45	86.4
Self-Exploration	3.06	0.70	0.26	−0.13	0.60	90.9
						
Client Codes						
No Code	77.77	11.34	0.82	0.62	0.92	-
APCS-NP Approach	4.35	3.32	0.77	0.52	0.90	-
APCS-NP Neutral	1.52	1.60	0.64	0.30	0.83	-
APCS-NP Avoid	0.98	1.13	0.64	0.30	0.83	-
APCS-P Approach	2.10	1.58	0.81	0.60	0.92	-
APCS-P Neutral	1.04	1.26	0.58	0.22	0.80	-
APCS-P Avoid	0.73	0.95	0.64	0.31	0.83	-
APCS-TB Approach	1.27	1.79	0.46	0.05	0.73	-
APCS-TB Neutral	5.25	2.76	0.81	0.60	0.92	-
APCS-TB Avoid	4.96	4.14	0.56	0.19	0.79	-
						
Outcome Variables						
MEDD	95.13	193.85	-			-
BPI—Pain Severity	5.57	1.93	-			-
BPI—Pain Interference	4.33	2.74	-			-
APCS-NP Goals	3.03	1.07	-			-
APCS-P Goals	1.25	0.94	-			-

Note: ICC = intraclass correlation coefficient; MEDD = morphine equivalent daily dose; BPI = Brief Pain Inventory; APCS = alternative pain care strategy; NP = non-pharmacological; P = pharmacological; TB = target behaviors. A total of 119 sessions, with 44 participants, and 22 sessions were double-coded. * The ICC for direction could not be calculated due to the negative average covariance among items.

**Table 2:** Correlations among proportions of within-session codes (n = 119).

Variable	1	2	3	4	5	6	7	8	9	10
APCS-NP Approach	-									
2. APCS-NP Neutral	0.45 ***	-								
3. APCS-NP Avoid	0.35 *	0.69 ***	-							
4. APCS-P Approach	0.18	0.13	0.21	-						
5. APCS-P Neutral	0.30 *	0.31 **	0.20	0.56 ***	-					
6. APCS-P Avoid	0.28	0.25	0.28	0.53 ***	0.38 *	-				
7. APCS-TB Approach	0.25	0.05	0.02	0.12	0.34 *	0.12	-			
8. APCS-TB Neutral	0.10	0.14	0.14	−0.13	0.17	0.04	0.66 ***	-		
9. APCS-TB Avoid	0.50 ***	0.21	−0.02	0.16	0.52 ***	0.21	0.57 ***	0.27	-	
10. No Code	−0.72 ***	−0.53 ***	−0.40 **	−0.38 *	−0.66 ***	−0.44 **	−0.66 ***	−0.51 ***	−0.80 ***	-

Note: * *p* < 0.05, ** *p* < 0.01, *** *p* < 0.001. APCS = alternative pain care strategy; NP = non-pharmacological; P = pharmacological; TB = target behavior; App = approach; Av = avoid; N = neutral.

## Data Availability

De-identified data are available from Brian Borsari upon request.
